# Risk Factors for Cervical Disc Arthroplasty Subsidence with Bryan Disc—A Retrospective Observational Analysis

**DOI:** 10.3390/jcm13061589

**Published:** 2024-03-10

**Authors:** Cheng-Ying Lee, Kuan-Kai Tung, Hsi-Kai Tsou, Wen-Hsien Chen, Chung-Yuh Tzeng, Ruei-Hong Lin, Tse-Yu Chen, Chih-Wei Huang, Ting-Hsien Kao

**Affiliations:** 1 Department of Neurosurgery, Taichung Veterans General Hospital, Taichung 40705, Taiwan; cklove5212@gmail.com (C.-Y.L.); ty761kimo@gmail.com (C.-W.H.); 2 Department of Orthopedics, Taichung Veterans General Hospital, Taichung 40705, Taiwan; david02200918@gmail.com (K.-K.T.); tcy0545@gmail.com (C.-Y.T.); 3 Functional Neurosurgery Division, Neurological Institute, Taichung Veterans General Hospital, Taichung 40705, Taiwan; foxboxccc@gmail.com; 4 Department of Rehabilitation, Jen-Teh Junior College of Medicine, Nursing and Management, Miaoli 356, Taiwan; 5 Department of Post-Baccalaureate Medicine, College of Medicine, National Chung Hsing University, Taichung 402202, Taiwan; chenws.tw@gmail.com; 6 College of Health, National Taichung University of Science and Technology, Taichung 403027, Taiwan; 7 Department of Radiology, Taichung Veterans General Hospital, Taichung 40705, Taiwan; 8 Department of Industrial Engineering and Enterprise Information, Tunghai University, Taichung 407705, Taiwan; 9 Department of Medicinal Botanicals and Foods on Health Applications, Da-Yeh University, Changhua 515006, Taiwan; 10 Institute of Biomedical Sciences, National Chung-Hsing University, Taichung 402202, Taiwan; 11 Ph.D. Program in Tissue Engineering and Regenerative Medicine, National Chung Hsing University, Taichung 402202, Taiwan

**Keywords:** cervical disc arthroplasty, implant subsidence, functional spinal unit height, Bryan disc, mean disc height, oversized, implant height

## Abstract

**Background**: Cervical disc arthroplasty (CDA) is currently used instead of fusion to preserve cervical spine motion. Cervical implant subsidence is a potential complication after CDA. **Methods**: Radiological measurements were recorded via patient anteroposterior and lateral radiographs in the neutral position. Subsidence was defined as a decrease of 3 mm or more in functional spinal unit height (FSUH) from which was measured on a post-operative (OP) radiograph. **Results**: This study included 104 patients who underwent 153 CDA levels with the Bryan Disc. Approximately one-quarter of the implants (22.9%) showed subsidence. Binary logistic regression analysis indicated that pre-OP mean disc height (DH) was identified as an independent risk factor for subsidence in multivariate analysis (0.151, 95% Confidence Interval 0–0.073, *p* = 0.018). Receiver operating characteristic curve analysis (area under the curve = 0.852, sensitivity 84.7%, specificity 77.1%) revealed a cut-off value of 4.48 mm for pre-OP Mean-DH in the risk for implant subsidence. **Conclusions**: In this study, the subsidence rate significantly increased when the implants were oversized beyond a pre-OP Mean-DH of approximately >4 mm. Moreover, the implant subsidence incidence was higher than that reported in previous studies. This is possibly due to endplate over-preparation or disc space over-distraction during placement at the same height as the Bryan Disc (8.5 mm).

## 1. Introduction

Since the 1950s [1], anterior cervical discectomy and fusion (ACDF) has been widely used as a surgical intervention for degenerative cervical spine disease [2]. However, while it effectively relieves the treated segment’s symptoms, it sacrifices segmental mobility, which can lead to adjacent segment degeneration and symptom recurrence [3,4,5,6]. To preserve functional spinal motion, cervical disc arthroplasty (CDA) with an artificial disc is the most common non-fusion technique used [7]. The benefits of CDA over traditional fusion surgery include maintaining the range of motion, reconstituting disc height and spinal alignment, avoiding adjacent segment degeneration (ASD), and preserving near-normal spine kinematics [7,8]. Some studies show a lower rate of reoperation and revision [9,10,11]. However, studies with a longer follow-up period after CDA have revealed more complications, such as metallosis, heterotopic ossification, and anterior bone loss [12,13,14,15,16,17]. Recently, implant subsidence has emerged as a rare but concerning CDA complication [14,15,18]. Although the axial stress in the neck is low, cervical artificial disc subsidence has still occurred in some cases during post-operative (post-OP) follow up.

Among fusion cases, as ACDF has been widely used since the late 20th century, post-OP cage subsidence has been well documented. The current literature suggests a subsidence rate ranging from 19.3% to 42.5% [19,20]. The risk factors for cage subsidence include: osteoporosis [21], endplate violation, small implant contact footprint [22], and the number of operative levels [23]. Unlike ACDF, the CDA experience and follow-up time are more limited, and the risk factors and mechanisms leading to implant subsidence remain uncertain. Radiographic subsidence rates in CDA widely varies from 0% to 33.3% in the literature, and few studies have sought to define the mechanisms of subsidence [24]. Although most radiographically detected implant subsidence is not clinically symptomatic, in rare cases, generally <3% in most large series [3,18], it can still lead to structural instability and disc space collapse, which may cause segmental kyphosis and contribute to ASD [25,26]. Due to the limited studies and the low overall subsidence rate, few published studies have defined the mechanisms of CDA subsidence.

This study retrospectively examined the subsidence risk factors in patients with cervical radiculopathy and/or myelopathy treated with CDA, exclusively using the Bryan Disc, and reviewed the existing ACDF and CDA subsidence literature.

## 2. Materials and Methods

### 2.1. Patient Population

Patients at a single institution who underwent CDA from June 2006 to September 2016 were enrolled in this study. All patients were operated on exclusively by a single neurosurgeon using the Bryan Disc (Medtronic Sofamor Danek, Memphis, TN, USA). All CDA were treated within the C3 to C7 disc segments. Patients who had previous cervical spine surgery, instability, history of infection, tumor etiology, or incomplete follow-up record were excluded. Demographic factors and operative clinical data, including age, gender, operative levels, implant placement segment, follow-up period, and smoking habits, were assessed. The relevant Institutional Review Board approved the study, which was conducted in accordance with the Declaration of Helsinki.

### 2.2. Radiographic Evaluation

**Figure 1:**
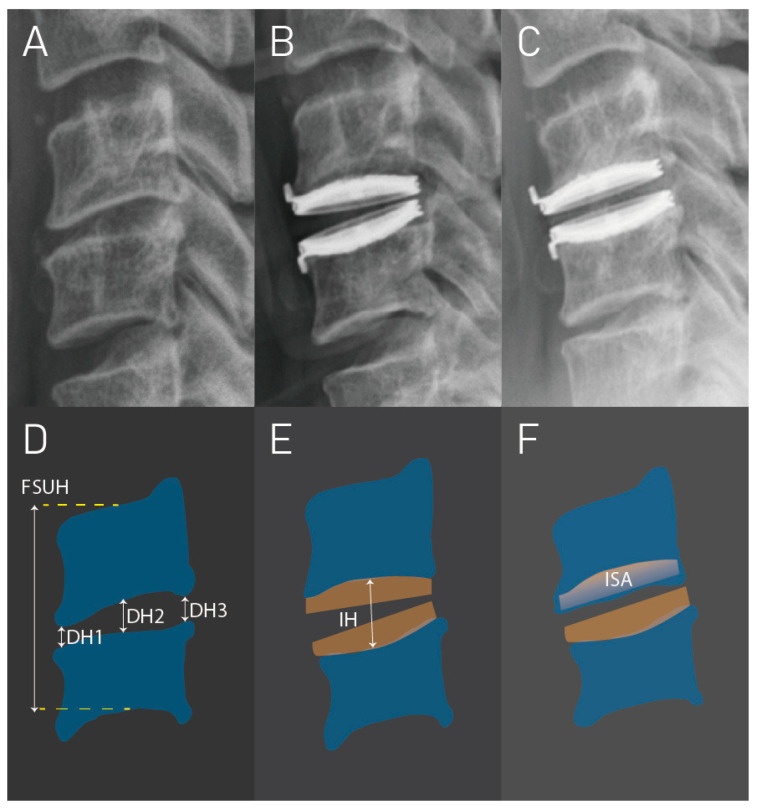
Grade 1 subsidence in a case that underwent C5–C6 cervical disc arthroplasty (CDA). (**A**–**C**) are the cervical radiographs before surgery, immediately after surgery, and at 1 year follow-up, respectively. (**D**–**F**), respectively, represent the schematic diagrams for radiographs (**A**–**C**). DH1 = anterior disc height; DH2 = middle disc height; DH3 = posterior disc height; FSUH = functional spinal unit height; IH = implant height; ISA = implant subsidence area.

**Figure 2:**
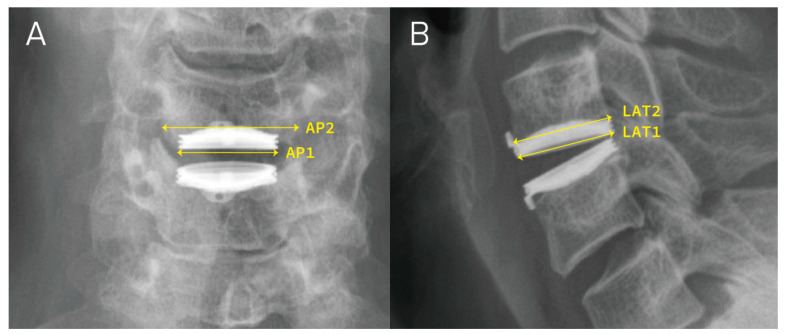
Illustration of measuring AP1, AP2, LAT1, LAT2. (**A**,**B**) represent the anteroposterior (AP) view and lateral (LAT) view of the post-operative radiographs, respectively. AP1 = the width of the Bryan Disc; AP2 = the width of the adjacent endplate; LAT1 = the length of the Bryan Disc; LAT2 = the length of the adjacent endplate.

The radiological measurements were conducted by two experienced neurosurgeons using anteroposterior (AP) and lateral (Lat) radiographs in the neutral position at four different time points: pre-operative (pre-OP), immediately post-operative (post-OP), one year follow-up, and last follow-up. The criterion for implant subsidence was a reduction ≧3 mm in the functional spinal unit height (FSUH) observed on the Lat radiograph [3,13,23]. The mean disc height (Mean-DH) prior to surgery was calculated as the mean of the anterior, middle, and posterior intervertebral disc spaces. The change in disc height (ΔDH) was obtained by measuring the difference between pre-OP Mean-DH and implant height (IH) (Figure 1). Additionally, we measured the ratio between the endplate length and the Bryan Disc on the AP and Lat radiographs (AP ratio and Lat ratio, respectively). The implant contact footprint between the Bryan Disc and endplate (CDA area) was determined by the sum of the AP and Lat ratios (Figure 2). Implant subsidence was graded as follows: Grade 0 for FSUH decrease < 3 mm (no subsidence), Grade 1 for FSUH decrease ≧ 3 mm but <5 mm (mild subsidence), Grade 2 for FSUH decrease ≧ 5 mm (moderate subsidence), and Grade 3 for FSUH decrease ≧ 5 mm with adjacent vertebral collapse (severe subsidence).

### 2.3. Calculation

All quantitative variables were reported as the mean with standard deviation, while the qualitative variables were presented as ratios and numbers. Continuous variables were evaluated using the paired Student’s *t*-test and one-way analysis of variance (ANOVA), whereas categorical variables were tested using the Pearson Chi-square test. Binary and multivariate logistic regression analyses were conducted to determine the odds ratio for subsidence. A *p*-value less than 0.05 was considered statistically significant. All statistical analysis was conducted using SPSS version 26 (IBM Corp., Armonk, NY, USA).

## 3. Results

Throughout the designated period of analysis, 125 patients met the eligibility criteria. However, seventeen were excluded from the study due to an insufficient follow-up period, while another four were excluded due to previous cervical spine surgery. Therefore, a total of 104 patients with 153 CDA operative segments utilizing the Bryan Disc were studied. The study cohort’s demographic data are presented in Table 1. All patients were diagnosed with either cervical radiculopathy and/or myelopathy. Radiological implant subsidence was detected in 35 implants (22.9%) in 18 patients (17.3%), with 33 cases (94%) of subsidence occurring at the vertebrae anterior edge, with a nearly equal distribution between the upper and lower vertebrae (52% and 48%, respectively). The mean follow-up duration of the cohort was 51 months, with a longer follow-up period observed in the subsidence group (71 vs. 43.5 months, *p* = 0.007). Half of the cohort comprised female patients. Eighty-three (54.2%) treatments were in the C5–C6 segment, with 55 (52.9%) patients receiving a single-level CDA. The pre-OP Mean-DH was smaller in the subsidence group (4.0 vs. 5.36 mm, *p* < 0.001). The ΔDH was higher in the subsidence group. The implant contact footprint did not display any statistical significance related to implant subsidence.

Binary logistic regression analysis revealed that pre-OP Mean-DH and ΔDH were both potential risk factors for implant subsidence in single variate models. However, the multivariate model identified only pre-OP Mean-DH as an independent risk factor for subsidence (0.151, 95% Confidence Interval 0–0.073, *p* = 0.018) (Table 2). Further analysis using receiver operating characteristic (ROC) curves was performed to determine the suitability of using pre-OP Mean-DH to predict subsidence risk. A cut-off value of 4.48 mm for pre-OP Mean-DH was established to reveal the risk of implant subsidence following CDA with the Bryan Disc (area under the curve = 0.852, sensitivity 84.7%, specificity 77.1%).

**Figure 3:**
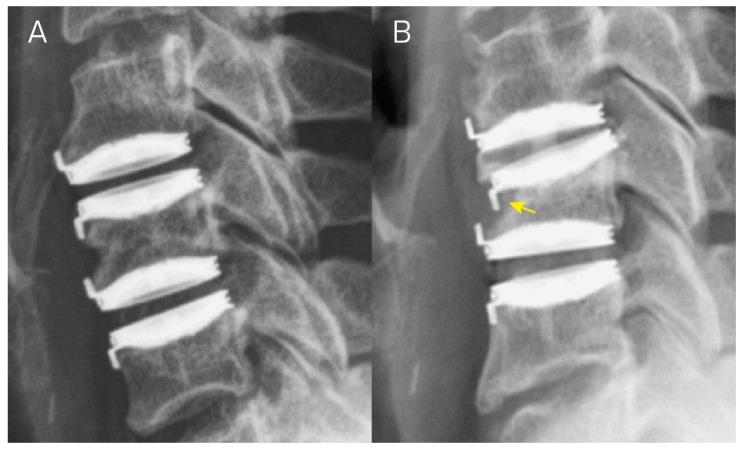
Lateral radiographs of a patient who underwent C4–C5 and C5–C6 cervical disc arthroplasty. (**A**,**B**) depict the lateral view of radiographs immediately after the surgery and at a 1-year follow-up, respectively. Panel (**B**) illustrates implant subsidence at the anterior upper edge of the C5 vertebra (yellow arrow), resulting in a decrease in functional spinal unit height of over 5 mm. This aligns with the criterion for Grade 2 subsidence.

The majority of cases (27 implants or 77%) exhibited Grade 1 subsidence. Grade 2 subsidence was observed in eight implants (Figure 3), with no patient meeting the criterion for Grade 3 subsidence.

## 4. Discussion

The Bryan Disc is a prosthetic device that imitates the natural movement of the cervical intervertebral disc in CDA. The device is composed of two convex titanium alloy spheres, an intervening polyurethane core that articulates with each sphere, a polyurethane circular wall, and a cavity [27]. It received US Food and Drug Administration (FDA) approval in 2005 for reconstructing the cervical disc following single-level discectomy for intractable radiculopathy and/or myelopathy in skeletally mature patients. The Bryan Disc was replaced by its second-generation product in 2016 and was discontinued in February 2020. The contraindications to implanting the Bryan Disc include active infection; allergy to titanium, polyurethane, or ethylene oxide residues; osteoporosis (defined as a dual-energy X-ray absorptiometry bone mineral density [BMD] T-score of −2.5 or worse); marked cervical instability on radiographs; significant kyphotic deformity; or significant lordosis reversal (Supplementary Materials).

We conducted a retrospective, longitudinal radiographic study to evaluate the change in FSUH at the target segments and the development of implant subsidence after at least 1 year of follow-up after CDA with the Bryan Disc. Furthermore, we analyzed potential risk factors for implant subsidence, including oversized implant, small implant contact footprint, age, gender, operative levels, implant placement segment, follow-up period, and smoking habit. Finally, we examined the correlation between these radiological findings and clinical outcomes to provide context for the results.

In contrast to fusion surgery, CDA offers a certain degree of mobility to the cervical spine after surgery, increasing the complexity of the biodynamics affecting bone growth and resorption. In this passive movable system, the artificial disc force distribution is variable and changes with different cervical postures. According to the pressure formula, the pressure exerted by an implant on the vertebral endplate is directly proportional to the force applied and inversely proportional to the surface area. These two factors in the pressure formula correspond to the two risk factors, namely implant oversizing and small implant contact footprint. Godlewski et al. reported that implant subsidence is significantly related to the coefficient value that represents the surface area ratio of the implant to the adjacent vertebral body bone surface areas. In their study, subsidence occurred significantly less frequently for coefficient values ≥ 0.37 [22]. This finding reinforces the significance of the surface area in the pressure formula. Nevertheless, in our investigation, we utilized the sum of the AP ratio and the Lat ratio as a metric to estimate the presumed Bryan Disc contact footprint. This metric did not differ significantly between the two cohorts (*p* = 0.18).

Data analysis revealed that patients with a subsided implant had a decreased FSUH at follow-up compared to the measurement taken immediately after the surgery. However, this decrease was similar to the pre-OP measurement, indicating that the FSUH tends to return to its natural height. This finding raises the question of whether exceeding the natural intervertebral disc height increases the risk of implant subsidence. Binary logistic regression revealed that pre-OP Mean-DH and ΔDH were potential risk factors for implant subsidence in single variate models. These two factors suggest that the gap between the pre-OP disc height and the implant height is responsible for implant subsidence. A smaller pre-OP Mean-DH or a higher post-OP disc space both contribute to increased pressure on the endplates. However, the multivariate model identified only pre-OP Mean-DH as an independent risk factor for subsidence. All patients in the study received the same brand of artificial disc, the Bryan Disc, which has a unique height of 8.5 mm. This standardization caused operative segments with a lower pre-OP Mean-DH to be propped up higher after the artificial disc was implanted. The study found that the subsidence rate significantly increased in segments with a pre-OP Mean-DH which was less than 4.48 mm. Therefore, placing an oversized artificial disc approximately 4 mm higher than the pre-OP Mean-DH is a risk factor for post-OP implant subsidence.

In lumbar disc arthroplasty, the possibility of subsidence is acknowledged as a potential complication [28], with reported device-related complication rates ranging from 0 to 6.5% [29,30]. As the cervical spine experiences less axial stress than the lumbar spine, cervical artificial disc subsidence is often deemed to be extremely rare. Nonetheless, even though 22.9% of implants in our study had radiological subsidence during follow-up, all were mild-to-moderate subsidence (100%) and these cases did not develop any new symptoms that required reoperation due to implant subsidence. This result was consistent with the findings from a previous publication on patients receiving ACDF [23]. Therefore, since implant subsidence is so easily overlooked, we suspect that there may be more occurrences of implant subsidence than we have clinically observed. At present, implant subsidence is merely a change in imaging and carries no clinical significance. However, the artificial disc has been in use for less than 30 years. The question remains whether current radiographic implant subsidence will progress to further clinical symptoms that require reoperation in the future. 

Kao et al. reported that undergoing surgery on multiple levels is a risk factor for implant subsidence in ACDF [23]. Since the US FDA has approved the implantation of artificial discs only for a maximum of two levels, we examined whether patients who underwent surgery on two levels were more likely to experience implant subsidence than those who underwent surgery on one level. Although our results indicated a higher proportion of subsidence in the two-level group than the one-level group (20% vs. 14.5%, respectively), the association between two-level surgery and implant subsidence was not statistically significant (*p* = 0.957). There was also no significant difference by implant placement segment. Whether patients who undergo surgery on more than two levels of CDA or who receive a combination of CDA and fusion surgery are at an increased risk of implant subsidence requires further investigation through the inclusion of more hybrid cases in future studies.

**Figure 4:**
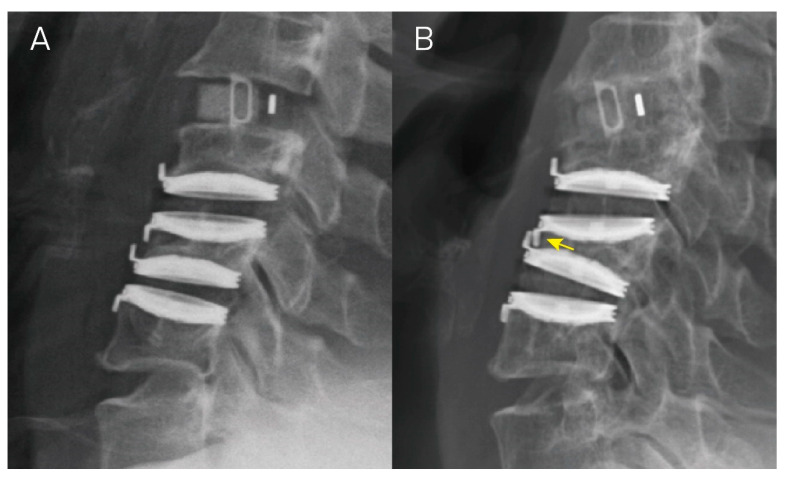
A hybrid case underwent anterior cervical discectomy and fusion at C3–C4 and cervical disc arthroplasty at levels C4–C5 and C5–C6. Panels (**A**,**B**) illustrate the lateral view of radiographs taken right after the operation and at a 1-year follow up, respectively. In Panel (**B**), there is an evident functional spinal unit height decrease > 5 mm at the C4–C5 and C5–C6 levels, accompanied by C5 vertebral collapse, resulting in Grade 3 subsidence in both segments (yellow arrow).

We defined Grade 3 subsidence as an FSUH decrease ≧ 5 mm with adjacent vertebral body collapse. When both the upper and lower endplates of a vertebra are severely subsided, leading to a reduction in its vertebral height, we refer to this condition as “body collapse”. In this particular case series, not one operative segment met this criterion. However, among other hybrid cases, a greater number of patients meeting the criterion for Grade 3 subsidence were observed (Figure 4). Moving forward, we aspire to analyze hybrid cases in order to characterize how adjacent segment fusion influences implant subsidence.

Osteoporosis is also recognized as a potential risk factor for implant subsidence. As the FDA considers osteoporosis a contraindication for CDA, we aimed to mitigate the potential impact of BMD on implant subsidence. All female patients over 50 years of age and male patients over 60 years of age underwent routine BMD testing before surgery, and patients with a T-score ≦ −2.5 were deemed ineligible for surgery. However, data on BMD were not consistently collected in younger patients, preventing us from examining the association between BMD and implant subsidence. When we analyzed BMD data from 32 patients above the aforementioned age thresholds in our study, we found no statistically significant differences in implant subsidence by BMD and T-scores (*p* = 0.072 and 0.063, respectively). This finding suggests that, so long as it is above a certain threshold, BMD will not affect implant subsidence incidence in patients. However, since we did not collect BMD data for all patients, this result cannot fully elucidate the relationship between osteopenia and subsidence. Therefore, we suggest that, if clinically feasible, all patients undergoing CDA surgery should undergo routine BMD testing, even young or middle-aged ones. This practice would facilitate more detailed and comprehensive analyses, as well as future studies on the impact of BMD on subsidence. Lee et al. reported that patients with a T-score between −1.5 and −2.0 may be included for disc arthroplasty only when they can receive a large sized prosthesis with prosthetic endplates greater than 10.5 cm^2^ [31], suggesting that increasing the footprint between the implant and the endplate correlates with the above concept of the pressure formula.

The current study has certain constraints that need to be acknowledged. In this series, the subsidence group had a longer follow-up time than the non-subsidence group did. This difference may reflect an operational bias caused by clinical practice. During post-OP follow-up in the outpatient clinic, patients found to have subsided implants were usually followed over time in order to track for any newly developed symptoms. Those patients without post-OP complications are often lost to follow-up after a few years. Furthermore, in this series, all implant subsidence occurred within one year after surgery (average 5.9 months). Therefore, the statistical difference does not affect the conclusions drawn in this study when excluding cases tracked for less than one year. However, Hacker et al. reported that subsidence has been reported in patients up to 4 years after CDA [15], so further research is needed to clarify the relationship between follow-up time and implant subsidence. This study did not include functional measures such as the Visual Analogue Scale in the statistical analysis. Additional research is necessary to further clarify whether implant subsidence impacts functional outcomes. 

In our study, 35 implants (22.9%) in 18 patients (17.3%) presented radiological implant subsidence, an incidence rate higher than that reported in the previous literature. This higher rate may be due to excessive endplate violation during Bryan Disc implantation. During endplate preparation, there is a potential risk of excessive milling and burring at the posterior endplate when removing the posterior bone spurs. This action is undertaken to effectively decompress the spinal cord and nerve roots. As a result, more compressive pressure may be exerted onto the implant anterior edge than to the posterior portion, leading to anterior subsidence. In our series, 33 cases (94%) of subsidence occurred at the anterior edge of the vertebrae, which may indirectly support the overmilling and burring hypothesis. However, as this study did not include artificial discs other than the Bryan Disc, and further comparisons with other artificial discs are needed to draw further conclusions.

The risk of implant subsidence is equal in significance to the potential for adjacent disc disease, representing critical considerations in the strategic planning of CDA. These factors are of paramount importance, not only in choosing between anterior and posterior surgical approaches, but also in the broader context of ensuring optimal spinal health. The preservation or restoration of sagittal vertical alignment is a pivotal goal that informs our decision-making process [32]. When evaluating the best surgical route, we meticulously weigh the implications of subsidence, which can lead to implant misalignment and compromise spinal stability, against the consequences of adjacent disc disease, which may precipitate further degenerative changes. Both scenarios could significantly impact patient outcomes, highlighting the necessity of a balanced and informed approach in surgical planning. This comprehensive evaluation underscores the importance of a holistic view in addressing spinal conditions, aiming to achieve the best possible balance between mechanical stability and biological health of the spine.

## 5. Conclusions

The Bryan Disc is associated with a high subsidence rate due to the excessive endplate preparation and disc space over-distraction intrinsically related to the insertion technique required for the placement of this CDA implant. In this study, the implant subsidence rate was higher than in previous reports, and significantly increased when oversized Bryan Discs were used, causing an increase of more than 4 mm in the pre-OP Mean-DH. While new artificial discs have been developed that require less endplate preparation and provide various-sized implants, appropriate size selection remains crucial. Future designs should consider subdivided size selection and reduced minimum size for patients with a narrow pre-OP intervertebral disc height.

## Figures and Tables

**Table 1:** Study population demographic data stratified by presence of subsidence.

	No Subsidence		Subsidence		Total		*p* Value
Participants	86		18		104		
CDA numbers, *n*	118		35		153		
Sex							1.000
Female	43	(50.0%)	9	(50.0%)	52	(50.0%)	
Male	43	(50.0%)	9	(50.0%)	52	(50.0%)	
Age, years	49.00	(43.00, 54.25)	49.00	(43.25, 55.00)	49.00	(43.00, 54.75)	0.976
Smoking, n	26	(30.2%)	5	(27.8%)	31	(29.8%)	1.000
Follow-up period, months	43.50	(16.50, 75.75)	71.00	(48.00, 135.50)	51.00	(20.75, 89.75)	0.007 **
Operative levels							0.597
Single level cases	47	(54.7%)	8	(44.4%)	55	(52.9%)	
Two level cases	39	(45.3%)	10	(55.6%)	49	(47.1%)	
Segments							0.594
Single level	47	(37.3%)	8	(29.6%)	55	(35.9%)	
Two level	79	(62.7%)	19	(70.4%)	98	(64.1%)	
C3–C4	11	(8.7%)	2	(7.4%)	13	(8.5%)	1.000
C4–C5	38	(30.2%)	6	(22.2%)	44	(28.8%)	0.553
C5–C6	68	(54.0%)	15	(55.6%)	83	(54.2%)	1.000
C6–C7	9	(7.1%)	4	(14.8%)	13	(8.5%)	0.247
pre-OP Mean-DH	5.36	(5.19, 5.52)	4.0	(3.7, 4.3)	5.05	(4.88, 5.22)	<0.001 **
ΔAP, mm	0.17	(0.15, 0.19)	0.21	(0.16, 0.25)	0.18	(0.16, 0.19)	0.056
ΔLat, mm	0.14	(0.12, 0.16)	0.14	(0.1, 0.19)	0.14	(0.12, 0.16)	0.970
ΔDH, mm	0.59	(0.57, 0.61)	0.67	(0.62, 0.72)	0.61	(0.59, 0.63)	0.002 **
AP ratio	0.92	(0.91, 0.92)	0.9	(0.88, 0.92)	0.91	(0.91, 0.92)	0.075
Lat ratio	0.94	(0.93, 0.95)	0.94	(0.92, 0.96)	0.94	(0.93, 0.95)	0.917
BMD	1.136	(1.09, 1.16)	0.998	(0.96, 1.03)	1.13	(1.05, 1.15)	0.072
T score	0.3	(0.05, 0.35)	−1	(−1.21, −0.75)	0.1	(−0.6, 0.3)	0.064

CDA, cervical disc arthroplasty; pre-OP, pre-operation; DH, disc height; AP, anteroposterior; Lat, lateral; ΔAP, difference in width between endplate and CDA in AP view; ΔLat, difference in width between endplate and CDA in Lat view; ΔDH, difference in height before and after CDA in Lat view; BMD, bone mineral density. Brackets: Median (Interquartile Range). ** *p* < 0.01.

**Table 2:** Bryan Disc subsidence independent risk factors identified by logistic regression.

	Simple Regression			Multiple Regression (Forward)		
	OR	95% CI	*p* Value	OR	95% CI	*p* Value
Age	1.003	(0.948, 1.061)	0.916			
Gender	1.127	(0.432, 2.938)	0.807			
Smoking	0.814	(0.292, 2.265)	0.693			
pre-OP Mean-DH	0.186	(0.103, 0.336)	<0.001 **	0.151	(0.059, 0.391)	<0.001 **
ΔAP-mm	35.557	(0.862, 1467.464)	0.06			
ΔLat-mm	1.059	(0.056, 20.097)	0.969			
ΔDH-mm	2.616	(1.707, 4.011)	<0.001 **	0.797	(0.395, 1.61)	0.528
AP ratio	0.001	(0, 2.301)	0.078			
Lat ratio	0.66	(0, 1470.868)	0.916			
Contact footprint of implant	0.016	(0, 6.824)	0.18			

*p*-Value < 0.05 was considered statistically significant. The 95% CI range is provided in brackets. ** *p* < 0.01. OR, odds ratio; CI, Confidence Interval; pre-OP, pre-operation; DH, disc height; CDA, cervical disc arthroplasty; ΔAP, difference in width between endplate and CDA in AP view; ΔLat, difference in width between endplate and CDA in Lat view; ΔDH, difference in height before and after CDA in Lat view; AP, anteroposterior; Lat, lateral.

## Data Availability

All data are available upon reasonable request from the corresponding author.

## Supplementary Materials

The following supporting information was accessed on 14 January 2024 and can be downloaded at: https://www.accessdata.fda.gov/cdrh_docs/pdf6/p060023c.pdf.
